# Human acute Chagas disease: changes in factor VII, activated protein C and hepatic enzymes from patients of oral outbreaks in Pará State (Brazilian Amazon)

**DOI:** 10.1590/0074-02760190364

**Published:** 2020-02-27

**Authors:** Valéria Regina Cavalcante dos Santos, Dina Antunes, Dilma do Socorro Moraes de Souza, Otacilio Cruz Moreira, Igor Campos de Almeida Lima, Désio A Farias-de-Oliveira, João Pedro Lobo, Ernesto de Meis, José Rodrigues Coura, Wilson Savino, Angela Cristina Verissimo Junqueira, Juliana de Meis

**Affiliations:** 1 Fundação Oswaldo Cruz-Fiocruz, Instituto Oswaldo Cruz, Laboratório de Pesquisas sobre o Timo, Rio de Janeiro, RJ, Brasil; 2 Secretaria de Saúde Pública do Estado do Pará, Belém, PA, Brasil; 3 Instituto Nacional de Ciência e Tecnologia em Neuroimunomodulação, Rio de Janeiro, RJ, Brasil; 4 Universidade Federal do Pará, Faculdade de Medicina, Belém, PA, Brasil; 5 Fundação Oswaldo Cruz-Fiocruz, Instituto Oswaldo Cruz, Laboratório de Biologia Molecular e Doenças Endêmicas, Rio de Janeiro, RJ, Brasil; 6 Universidade do Estado do Rio de Janeiro, Departamento de Estatística, Rio de Janeiro, RJ, Brasil; 7 Fundação Oswaldo Cruz-Fiocruz, Instituto Oswaldo Cruz, Laboratório de Doenças Parasitárias, Rio de Janeiro, RJ, Brasil; 8 Instituto Nacional de Câncer, Rio de Janeiro, RJ, Brasil

**Keywords:** Chagas disease, oral transmission, liver enzymes, coagulation factors, parasite load

## Abstract

Oral transmission of Chagas disease has been increasing in Latin American countries. The present study aimed to investigate changes in hepatic function, coagulation factor levels and parasite load in human acute Chagas disease (ACD) secondary to oral *Trypanosoma cruzi* transmission. Clinical and epidemiological findings of 102 infected individuals attended in the State of Pará from October 2013 to February 2016 were included. The most common symptoms were fever (98%), asthenia (83.3%), face and limb edema (80.4%), headache (74.5%) and myalgia (72.5%). The hepatic enzymes alanine aminotransferase (ALT) and aspartate aminotransferase (AST) of 30 ACD patients were higher compared with controls, and this increase was independent of the treatment with benznidazole. Moreover, ACD individuals had higher plasma levels of activated protein C and lower levels of factor VII of the coagulation cascade. Patients with the highest parasite load had also the most increased transaminase levels. Also, ALT and AST were associated moderately (r = 0.429) and strongly (r = 0.595) with parasite load respectively. In conclusion, the present study raises the possibility that a disturbance in coagulation and hepatic function may be linked to human ACD.

*Trypanosoma cruzi*, the causative agent of Chagas disease is a eukaryotic parasite of the family *Trypanosomatidae*. The World Health Organization estimates that 8 million people are infected worldwide causing more than 10,000 deaths/year, mostly in Latin America.^1^ Starting in the 1980s, attention was drawn to the globalisation of Chagas disease due to travel and migration movements from Latin America to the United States, Europe, Asia and Oceania.^1^ ^,^ ^2^ Disease can be transmitted by insect vectors, blood transfusion, organ transplantation, orally and congenitally.^3^ The first probable oral outbreak of human acute Chagas disease (ACD) in Brazil was reported in 1968, in Rio Grande do Sul State, involving 18 patients with six deaths.^3^ Similarly, 19 cases occurred in the State of Santa Catarina in 2005, with three deaths reported.^4^ The largest outbreak of *T. cruzi* infection by oral transmission involved 103 cases of ACD occurred in Venezuela, with an international impact.^5^ Presently, almost 70% of ACD cases in Brazil are associated to consumption of *T. cruzi* contaminated food.^6^ The State of Pará, in the Brazilian Amazonian region, is the most affected region, with 2,030 out of the 16,807 reported cases in the period of 2000-2016.^7^

In respect to the pathophysiology of human ACD, haematological and biochemical changes have been described, such as anaemia and thrombocytopenia.^8^ ^,^ ^9^ Haemorrhagic manifestations and severe gastritis have also been reported and may cause death.^10^ Hepatic alterations are also common in patients with ACD and may manifest as hepatomegaly, jaundice and elevated liver enzymes, aspartate and alanine transaminases.^(4,9)^ Nevertheless, it is not known if blood coagulation proteins, which are synthesised mainly by hepatocytes, are altered during human ACD. Some reports attempted to relate the pro-thrombotic and pro-inflammatory profile in patients with chronic Chagas disease, with hypercoagulability markers such as prothrombin fragment 1 + 2 (F_1+2_), D-dimer, plasminogen activator inhibitor type 1 and fibrinogen were higher compared to healthy volunteers, in addition to increased levels of serum IL-6.^11^ ^,^ ^12^ ^,^ ^13^ ^,^ ^14^ Herein, we evaluated the clinics, hepatic function, FVII, APC coagulation factor levels and parasite load of ACD patients in outbreaks of oral infection in the State of Pará from October 2013 to February 2016.

Here, we conducted a prospective case-control study involving ACD patients with epidemiological evidence of acute oral transmission (food as a likely source of contamination, simultaneous occurrence of more than one case with epidemiological linkage and without Romaña’s sign or chagoma of inoculation), assisted at the University Hospital João de Barros Barreto in the State of Pará. The individuals were included in the period October 2013 to February 2016. Diagnosis of ACD was confirmed by parasitological and conventional serological tests for *T. cruzi* detection [indirect immunofluorescence and enzyme-linked immunosorbent assay (ELISA)] in collaboration with the Central Laboratory (LACEN) of the State of Pará. All patients received treatment with antiparasitic medication Benznidazole (BZ) according to the protocol of the Brazilian Ministry of Health. Individuals in the chronic phase of the disease, clinical suspicion of co-infection or with cross-reactivity reaction were not included in our analyses. 

Plasma samples of 30 individuals without infection (negative serology), living in the same area, age and sex matched, were included as a control group; being kindly provided by the State of Pará blood bank (HEMOPA Foundation). The individuals recruited for this study did not receive any blood transfusion or organ transplantation prior to blood harvesting. The study was approved by the ethical committee of the Oswaldo Cruz Foundation (CAEE: 19248813.5.0000.5262) and followed the principles expressed in the Declaration of Helsinki. ACD patients and healthy individuals participated as volunteers and agreed to the “Terms of Free and Informed Consent” (signed informed consent was obtained from each subject). For each individual, 20 milliliters of venous blood were collected using BD Vacutainer® Plus Plastic EDTA K3 tubes and then centrifuged for 15 min at 1000 g at 4~8ºC in order to acquire plasma samples.

Coagulation factor levels in plasma were determined using the ELISA kits: FVII and APC (E-EL-H0758 and E-EL-H0453 respectively, Elabscience, China) according to the manufacturer’s instructions. All samples were measured by spectrophotometry, using the SpectraMax M2e device (Molecular Devices, Sunnyvale, CA, USA). Alanine aminotransferase (ALT) and aspartate aminotransferase (AST) were quantified in plasma samples using the Reflotron® Plus analyser (Roche Diagnostics, Mannheim, Germany) according to the manufacturer’s instructions. DNA extraction from plasma and quantitative duplex real-time polymerase chain reaction (qPCR) was performed from 200 μL of plasma samples, as previously described,^15^ using the QIAamp DNA Mini kit (Qiagen, Valencia, CA). The DNA eluate was stored at -20ºC until use in qPCR analysis. The qPCR reactions were carried out in a final volume of 20 μL containing 5 μL of DNA template, 10 µL of 2X TaqMan® Universal PCR Master Mix (Applied Biosystems, USA), 750 nM of cruzi1 (5′ASTCGGCTGATCGTTTTCGA3′) and cruzi2 (5′AATTCCTCCAAGCAGCGGATA3′) primers and 50 nM cruzi3 probe (5′FAM-CA CACACTGGACACCAA-NFQ-MGB3′) specific for the satellite region of the nuclear DNA of *T. cruzi*, 100 nM IAC Fw (5′CCGTCATGGAACAGCACGTA3′) and IAC Rv (5′CTCCCGCAACAAACCCTATAAAT 3′) primers and 50 nM IAC Tq probe (5′ VIC-AG CATCTGTTCTTGAAGGT-NFQ-MGB 3′) as an exogenous internal positive control of qPCR. Cycling conditions were a first step at 95ºC for 10 min, followed by 40 cycles at 95ºC for 15 s and 58ºC for 1 min. The amplifications were carried out in an ABI Prism 7500 Fast device (Applied Biosystems, USA). Standard calibration curves for plasma were constructed by serial dilution of DNA extracted from plasma samples obtained after whole blood spiking with *T. cruzi* epimastigotes (Dm28c), ranging from 10^5^ to 1 parasite equivalents per milliliter of blood (par. eq./mL). The GraphPad Prism 6 package (GraphPad Software Inc.) was used for the statistical analysis of the plasma levels of coagulation factors and hepatic enzymes (Fig. 1). Data were subjected to the D’Agostino-Pearson normality and Shapiro-Wilk tests to determine whether they were sampled from a Gaussian distribution. Since samples deviated from a Gaussian distribution, we applied so a non-parametric test (Mann-Whitney U test). Statistical significance was considered at p ≤ 0.05. Regarding the correlogram matrix and the principal component analysis (PCA) (Fig. 2), the software applied was R-software, version 3.4.4 (R Core Team, 2018). The R-software packages used were: pcaMethods,^16^ and corrplot.^17^

**Fig. 1:**
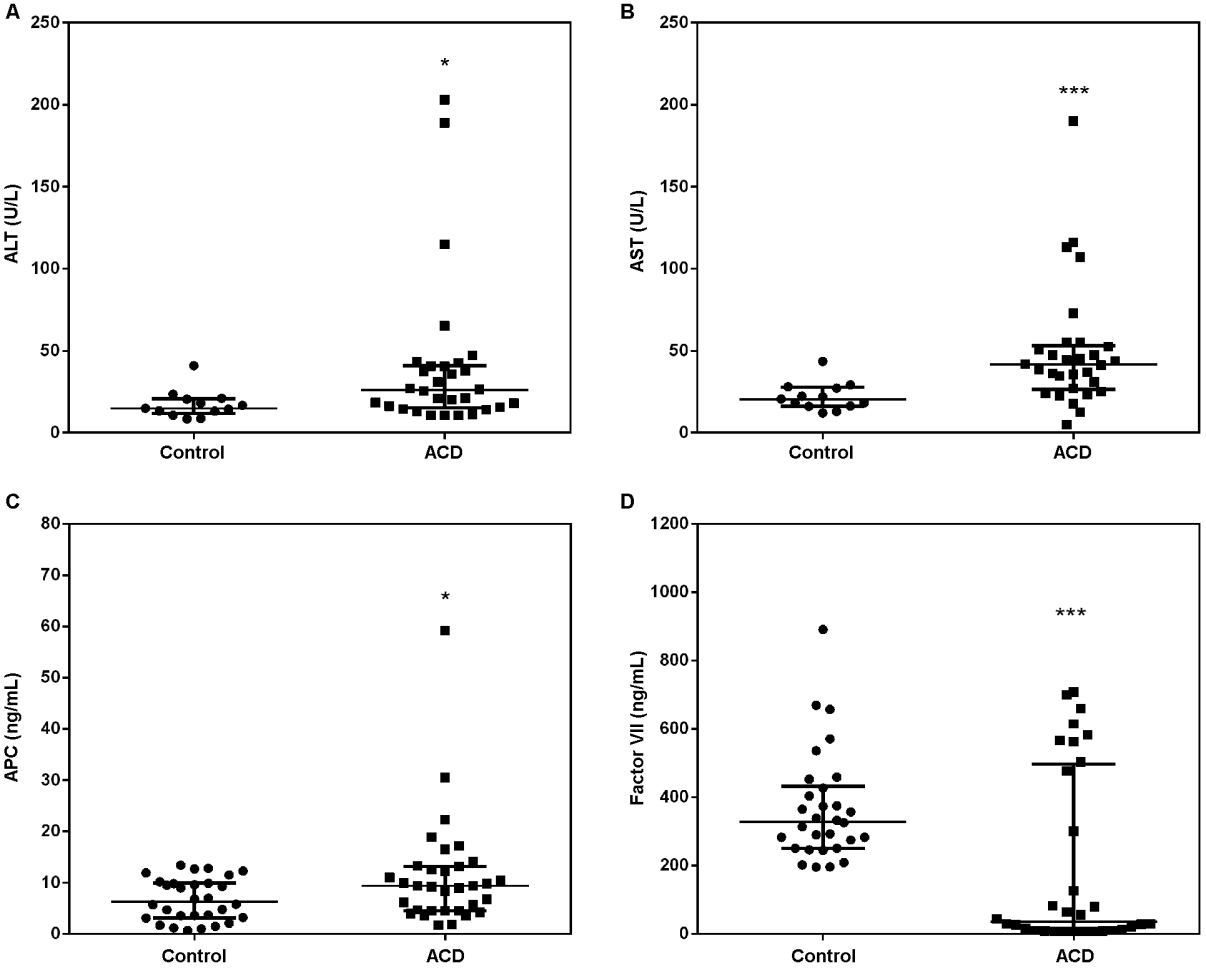
alterations in plasma levels of coagulation factors and hepatic enzymes in acute Chagas disease (ACD) patients. Panels A and B show the alanine aminotransferase (ALT) and aspartate aminotransferase (AST) levels, respectively. The group of acutely infected patients correspond to N = 30, 17 males and 13 females, with an average age of 39 years. Panel C depicts activated protein C (APC) levels from ACD patients. The group of acutely infected patients correspond to N = 33, 17 males and 16 females, with an average age of 39 years. Panel D shows data on the FVII coagulation factor. The ACD group correspond to N = 32, 17 males and 15 females, with an average age of 39 years. Control group for measurements of ALT and AST corresponds to N = 13, 2 males and 11 females, with an average age of 43 years. Control group for measurements of FVII and APC corresponds to N = 30, 14 males and 16 females, with an average age of 39 years. The bar represents the median with interquartile range of each group. * p < 0.05, ***p < 0.001.

**Fig. 2:**
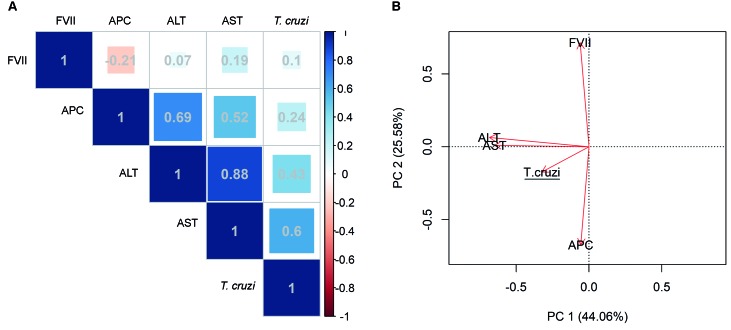
correlogram matrix of parasite load with coagulation factors FVII, activated protein C (APC) and hepatic enzymes alanine aminotransferase (ALT), aspartate aminotransferase (AST) in acute Chagas disease patients. A-Positive (blue) and negative (red) correlation levels are indicated at the scale bar. The colour scale illustrates the numerical value of the ratio. Numbers indicate the relationship between the two variables, where 1 indicates a perfect degree of association and -1 has opposite effects. B- The arrows indicate loadings of variables results. ALT x AST p = 0.002; AST x *T. cruzi* p = 0.059; APC x ALT p = 0.029; APC x AST p = 0.060; APC x FVII p = 0.694. N = 8, degree of freedom = 6.

The average age of the 102 ACD patients included in this study was 40 years, with 55% of the patients being males and 45% females. All individuals were diagnosed by parasitologic tests as bearing ACD. Patients came from 19 municipalities in the State of Pará, mostly from Abaetetuba 26.5% (n = 27), Belém 15.7% (n = 16) and Cametá 15.7% (n = 16). The most common symptoms were fever, asthenia, face and limb edema, headache and myalgia. Disturbances of ventricular repolarisation and tachycardia were also present. These findings are summarised in Table I.

**TABLE I:** Frequencies of signs and symptoms in 102 patients with acute Chagas disease in the State of Pará, Brazil

Signs and symptoms	N (%)
Fever	100 (98.0)
Asthenia	85 (83.3)
Facial edema	82 (80.4)
Headache	76 (74.5)
Myalgia	74 (72.5)
Abdominal pain	42 (41.2)
Dyspnea	38 (37.3)
Edema in legs	32 (31.4)
Tachycardia	25 (24.5)
Cough	8 (7.8)
Exanthema	6 (5.9)
Hepatomegaly	2 (1.96)
Hemorrhagic manifestations	2 (1.96)
Jaundice	1 (0.98)

In order to compare hepatic enzymes and coagulation plasma levels in this cohort, 30 to 33 patients were selected by their ages and proportional sex ratio to our analysis. Fig. 1 shows the changes in biomarkers of liver function (Fig. 1A-B) and coagulation factors (Fig. 1C-D) of ACD patients and healthy subjects. There was a significant increase in the levels of ALT and AST in plasma of ACD patients compared to healthy subjects.

It is well described that Benznidazole (BZ) provokes a broad spectrum of adverse effects including hepatotoxicity. Actually, most patients of the cohort were under BZ treatment at the time of sample collection (data not shown). Nevertheless, no significant differences of ALT and AST plasma levels were seen when BZ treated patients were compared with BZ untreated subjects [Supplementary data (Fig. 1)], suggesting that the elevated liver enzymes were associated with the infection itself.

Blood coagulation is mediated by factors such as FVII and controlled by anticoagulant proteins such as APC. Plasma levels of FVII and APC coagulation factor were analysed in these ACD patients. Circulating FVII levels were higher in healthy subjects as compared with their ACD countrparts. However, among acute patients, two different groups were evident: one with lower levels of FVII and another one that presented higher levels (Fig. 1). The two patients that have described mild symptoms of bleeding belonged to the FVII low level group. We demonstrated that APC levels were significantly increased in ACD patients, as compared to healthy controls (Fig. 1).

Previous studies^18^ showed that it is possible to quantify parasite load in serum samples without significant differences when comparing the results of parasite load from chronic Chagas disease patients peripheral blood samples. Here, for the first time, we performed a quantification of *T. cruzi* load in patient’s plasma samples by qPCR. One standard curve was constructed ranging from 10^5^ to 1 par. eq./mL with DNAs extracted from plasma derived from artificially contaminated blood. An exogenous internal amplification control (IAC) was used and its amplification produced results within the range to validate the results. The highest and lowest parasite load observed in ACD samples were 1843.8 ± 65.2 and 1.05 ± 0.26 par. eq./mL, respectively (Table II). Of note, only eight out of 30 plasmas from ACD1 patients presented measurable parasite load by qPCR. The possible loss of parasite detection of the parasite by qPCR observed in our study may be explained by the fractioning process of blood to plasma, especially in patients with lower circulating parasite counts.

**TABLE II:** Clinical summary comprising *Trypanosoma cruzi* load, coagulation factor levels, alanine aminotransferase (ALT) and aspartate aminotransferase (AST) alterations in eight patients with acute Chagas disease

Age (years)	Sex	Days of infection	Par. Eq./mL Mean ± SD	FVII (ng/mL)	APC (ng/mL)	ALT (U/L)	AST (U/L)
32	M	20	1.0 ± 0.2	660.30	14.1	37.4	41,2
27	F	12	4.5 ± 0.4	300,80	4.7	27.0	38,6
48	F	26	21.2 ± 6.1	80.20	11.0	37.8	37.0
58	M	26	35.6 ± 7.3	29.70	17.2	43.2	73.0
41	F	8	42.0 ± 7.0	566.17	12.5	5.6	27.3
32	F	18	109.7 ± 1.7	582.70	10.5	31.0	45.1
36	F	10	456.1 ± 58.4	698.85	13.1	203.0	116.0
36	M	Unknown	1843.8 ± 65.2	82.60	16.5	189.0	107.0

APC: activated protein C; SD: standard deviation.

The correlation of parasite load, coagulation factors and hepatic enzymes was performed after applying Shapiro-Wilk’s test and, then, Spearman’s correlation and can be seen in Fig. 2. The results revealed a very strong positive correlation of ALT and AST variables (r = 0.881), a strong positive relationship between AST and parasite load (r = 0.595) and APC with ALT and AST (r = 0.690 and r = 0.594), respectively. Moreover, weak inverse correlation between APC and FVII (r = -0.214) was observed. In this way, principal component analysis showed that three components accounted for 92.50% of the total variance explained. The first principal component accounted for 44.06% of the explained variance while the second and third corresponded to 25.58% and 22.86%, respectively. In the loadings plot for the first PC, the ASL and AST variables were almost overlapping, indicating a very strong positive correlation. Also, the parasite load (*T. cruzi*) was in the same direction, suggesting a correlation among those three variables. The second principal component showed that the variable FVII is in opposition to APC.

ACD caused by oral transmission has been increasingly reported in Brazil and other Latin American countries. Herein we reported a multiparametric analysis with clinical results of oral ACD patients from the State of Pará. Our epidemiological surveys showed that 98% of 102 patients ingested açaí in a daily basis, and this was the most likely source of transmission.^(7)^

Fever was experienced by nearly all patients followed by asthenia. In agreement with a previous study from the Pará State, the most common electrocardiographic change in ACD patients contaminated by oral transmission corresponded to alterations in ventricular repolarisation (72.6%); actually, we observed this disturbance in 84.3% of the patients.^19^

Haematological symptoms, including epistaxis, gum bleed and hemorrhagic manifestations were found in 2% of patients. In accordance, an outbreak of oral infection in the Northeast of Brazil, Ceará, hemorrhagic manifestations concomitant with high levels of transaminases were reported in three patients out of eight subjects.^(8)^ Interestingly, a recent study in mice demonstrated the involvement of the pro-inflammatory cytokine IL6 and hemostatic derangement in ACD. In this study, oral ACD induced low platelet count, increased bleeding and coagulation time, in parallel with high parasitaemia.^(19)^ Moreover, haematological changes occurred with prolonged activated partial thromboplastin time, Factor VIII consumption and increased D-dimer levels, suggesting signs of disseminated intravascular coagulation.^20^

In the present study gastrointestinal bleeding was not seen, thus differing from the symptoms observed in the outbreak in the State of Santa Catarina, southern Brazil, in March 2005 where three fatal cases were disclosed.^(4)^ Mortality rates in orally infected patients were higher (8-35%) when compared with the classical vectorial transmission route (< 5-10%).^(2)^ Nonetheless, no death was reported in this study.

We also observed changes in liver enzymes as well as a correlation between liver involvement with the parasite load, together with FVII and APC levels. We found that patients with highest parasite loads also showed highest ALT and AST levels supporting the hypothesis of liver involvement. Increase in ALT and AST levels can be highly suggestive of liver injury, although both enzymes are not liver-restricted, since they can be found in skeletal and cardiac muscles, brain and that some drugs enhance the corresponding gene expression. In this respect, previous studies have noted that treatment with BZ triggers multiple side effects in adults including hepatotoxicity.^21^ ^,^ ^22^ It has been described a slight increase in ALT and AST during the treatment with BZ in chronic patients of Chagas disease from 15 to 30.5U/L and 22.8 to 31.68U/L, respectively.^23^ In our study, patients underwent significantly changes in circulating contents of liver enzymes and these alterations were independent of the treatment since both BZ treated and BZ untreated groups exhibited high ALT and AST plasmatic levels and there were no statistical differences between them.

Liver alterations can interfere with factor VII^24^ and APC levels since both enzymes are vitamin K-dependently synthesised in the organ. Depletion of FVII has been associated with pathologic situations such as tumors, sepsis, antiphospholipid antibodies, aplastic anaemia and haematopoietic stem cell transplantation.^25^ Interestingly, in our study, the two patients that presented mild symptoms of bleeding such as nosebleeds, bleeding gums and heavy menstrual periods had low levels of FVII. The proteolytic enzyme APC is a normal plasma component, indicating that protein C is being activated *in vivo* continuously. In a previous study, comprising patients with venous thromboembolism (VTE), circulating plasma APC levels and APC/PC ratios were significantly higher in VTE patients than in healthy controls.^26^ Other findings revealed a correlation of endogenous acute coagulopathy (EAC) in severely injured and hypoperfused trauma patients with activation of the protein C pathway and increased morbidity and mortality.^27^ Furthermore, in an experimental mouse model of EAC with trauma and significant hemorrhagic shock, it was showed increased levels of APC (2.30 *versus* 13.58 ng/mL) in comparison with control mice.^28^ Herein, we demonstrated that APC levels were significantly higher in ACD patients.

The principal component analysis (PCA), a method used to design multivariate data in a smaller size space, without the relationship between the samples being unaffected.^29^ This compression is done through linear combinations of variables, resulting in new variables, called principal components, PCs. In the occurrence of missing values in the original data matrix, an alternative is the use of probabilistic PCA.^30^

In our study, it has the advantage of being a tool to visualise and discover relationships between our five distinct variables (ALT, AST, *T. cruzi*, FVII and APC) in a bi-dimensional space without affecting the real sample relationship, thus detecting atypical behavior samples. We showed that ASL and AST were almost overlapped, indicating a strong positive correlation, which was expected since both enzymes are released in acute hepatocellular injury. Also, the parasite load was in the same direction, suggesting a correlation between those three variables, meaning that acute liver injury occurring in ACD depends on the *T. cruzi* burden.

Finally, the present study provides evidence that a disturbance in coagulation and hepatic function is linked to human acute Chagas disease.
