# Supplementary material for: Human acute Chagas disease: changes in factor VII, activated protein C and hepatic enzymes from patients of oral outbreaks in Pará State (Brazilian Amazon)
Source: Mem Inst Oswaldo Cruz. 2020 Feb 27;115:e190364. doi: 10.1590/0074-02760190364 (PMC7046146; doi:10.1590/0074-02760190364)
Supplement: Supplementary file 1 [file 1678-8060-mioc-115-e190364-s.pdf]

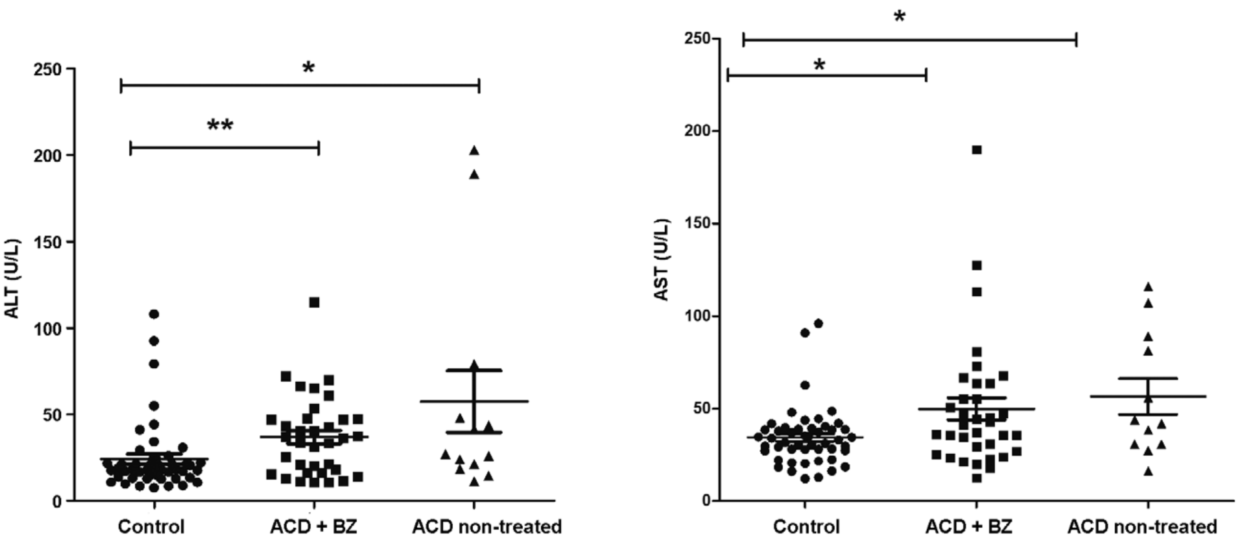

Hepatic enzymes in Benznidazole treated and non-treated acute Chagas disease (ACD) patients. Panels show the alanine aminotransferase (ALT) and aspartate aminotransferase (AST) levels respectively in control, Benznidazole (Bz, 5 to 7 mg/Kg) treated and non-treated ACD patients. Mean of Bz treatment time was 2-7 days. ALT- The control group corresponded to N = 49, 24 males and 25 females, with an average age of 39 years. ACD plus Bz group N = 35, 18 males and 17 females; ACD non-treated group N = 13; seven males and six females. The average age of 38.5 years. AST- The control group corresponds to N = 49, 24 males and 25 females, with an average age of 39 years. ACD plus Bz group N = 35, 18 males and 17 females; ACD non-treated group N = 11, seven males and five females. The average age of 38.5 years. Statistical analyses were carried out with two way Anova test. The bar represents the mean with standard error (SE) of each group. * $p < 0.05$, ** $p < 0.01$.

CC BY
